# Supplementary material for: Early Life Stress and High FKBP5 Interact to Increase Anxiety-Like Symptoms through Altered AKT Signaling in the Dorsal Hippocampus
Source: Int J Mol Sci. 2019 Jun 4;20(11):2738. doi: 10.3390/ijms20112738 (PMC6600369; doi:10.3390/ijms20112738)
Supplement: Supplementary file 1 [file ijms-20-02738-s001.pdf]

Supplementary Material

Supplementary Figures

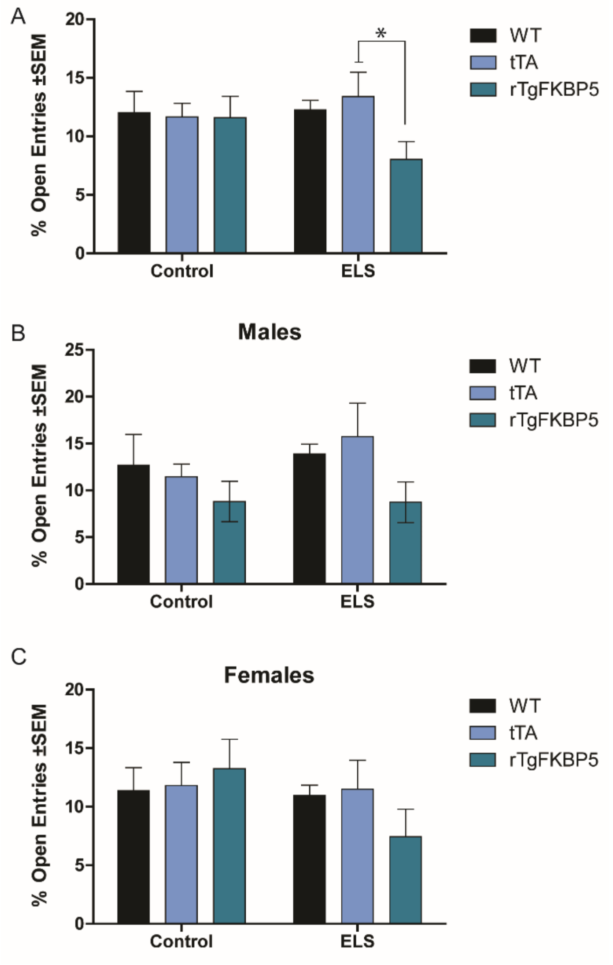

Supplementary Figure S1. FKBP5 overexpression combines with ELS to increase anxiety-like behavior. (**A**) Percent of open arm entries as determined by the number of open arm entries divided by the total entries into any quadrant ± SEM. Specific data from (**B**) Male and (**C**) Female mice is shown. Significant results were considered when *$p < 0.05$. ELS = early life.

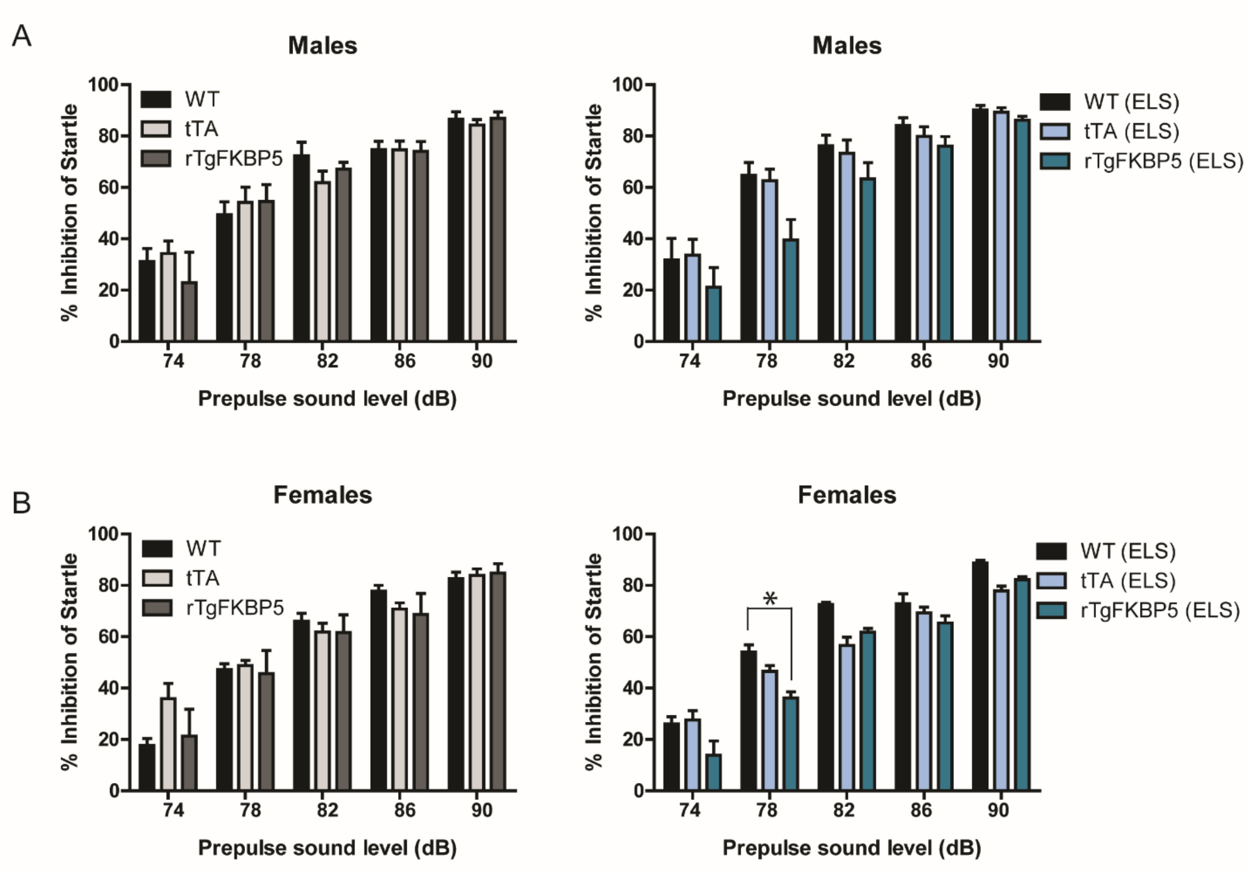

Supplementary Figure S2. Sex differences did not influence prepulse inhibition in rTgFKBP5 mice exposed to stress. Startle response as a percentage of prepulse inibition to 74, 78, 82, 86, and 90 dB stimulus from **Figure 2** broken down into (**A**) male or (**B**) female mice. Data is represented as standard error of the mean (SEM) and analyzed by two-way ANOVA (see **Table 1** for analysis). Significant results were considered when $*p < 0.05$. ELS = early life.

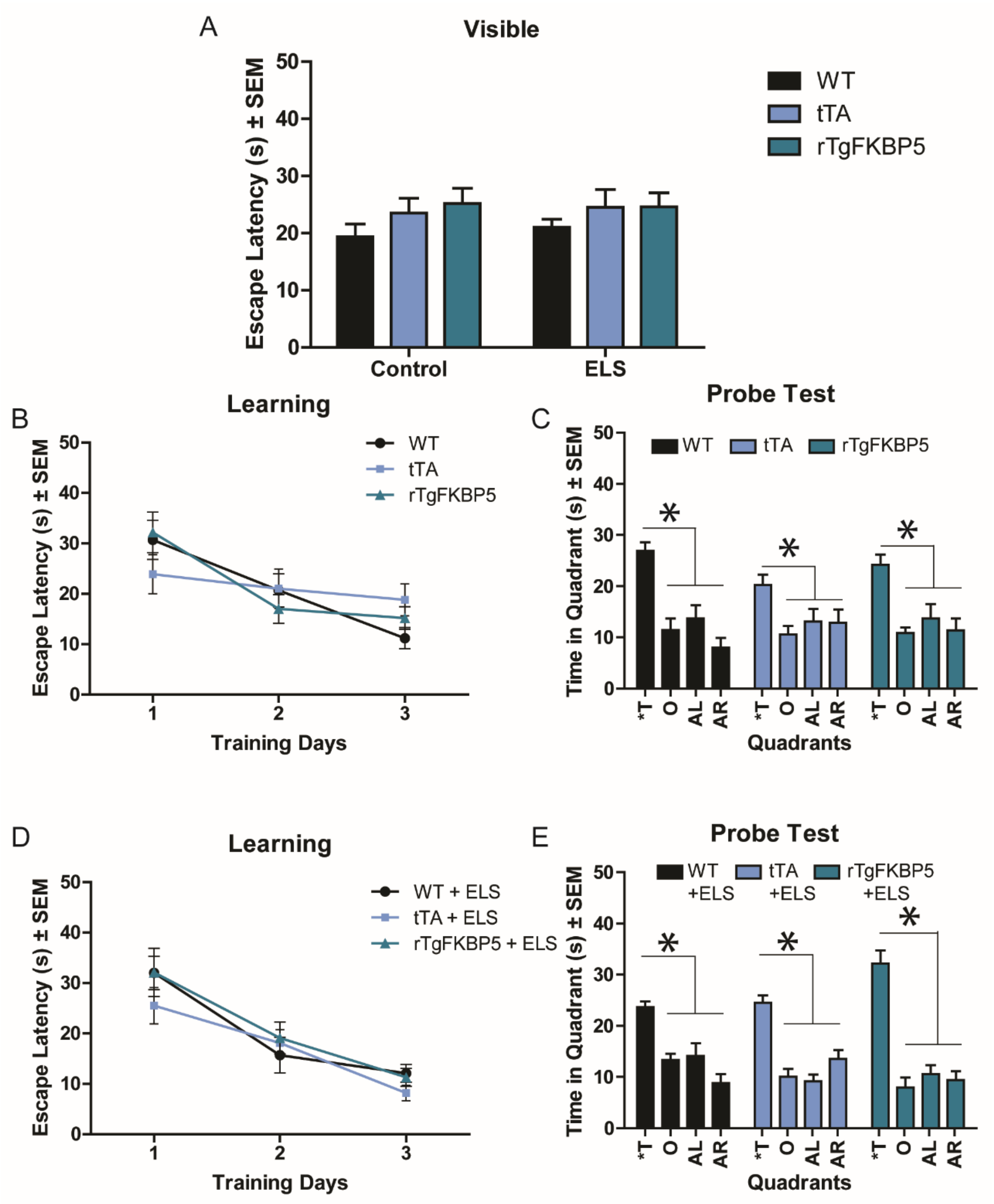

Supplementary Figure S3. Spatial learning is not affected by high levels of FKBP5 or stress. (**A**) Escape latency for finding the visible platform on day 1. Spatial learning and memory analysis of non-stressed mice (WT= 11, tTA =10, TgFKBP5 = 9) during (**C**) training days and (**C**) probe test using the Morris Water Maze (MWM) task. Data analysis for spatial learning and memory of early stressed mice (WT= 10, tTA = 10, rTgFKBP5 = 10) during (**D**) training days and (**E**) probe test. Each training day represents a block of 4-60 seconds trials. D = day, s = seconds, ELS = early life stress; Quadrants: T = target, O = Opposite, AR = adjacent right, AL = adjacent left. SEM = standard error of the mean. Data were analyzed by two-way ANOVA where significant results are represented as * = $p < 0.05$. Refer to **Figure 3** for spatial reversal learning test.
